# Detection of *Colpodella* sp. in blood of an ICU patient in Guizhou Province, China: a preliminary molecular report

**DOI:** 10.3389/fmed.2025.1638864

**Published:** 2025-10-01

**Authors:** Xiaopeng Yang, Yisong Dai, Jiashun Yu, Jixia Tang, Xingxing Chen, Qiu Chen, Wuchun Cao, Jiahong Wu, Fuxun Yu, Lin Zhan

**Affiliations:** ^1^School of Public Health, The Key Laboratory of Environmental Pollution Monitoring and Disease Control, Ministry of Education, Guizhou Medical University, Guiyang, China; ^2^School of Public Health, Guizhou University of Traditional Chinese Medicine, Guiyang, Guizhou, China; ^3^School of Public Health, Zunyi Medical University, Zunyi, Guizhou, China; ^4^Beijing Institute of Microbiology and Epidemiology, Academy of Military Medical Sciences, Beijing, China; ^5^NHC Key Laboratory of Pulmonary Immune-Related Diseases, Guizhou Provincial People’s Hospital, Guiyang, China

**Keywords:** *Colpodella* sp., Guizhou, protozoan, tick-borne pathogen, 18S rRNA

## Abstract

*Colpodella* sp. is an emerging tick-borne protozoan that causes fever, anemia, and neurological symptoms. Although this protozoan has previously been identified in animals and ectoparasite vectors within Guizhou Province, its prevalence among human populations remains unclear. We report a case of *Colpodella* sp. infection in a patient admitted to the intensive care unit (ICU) of a tertiary hospital in Guizhou Province, China. This case represents the first documented human infection with *Colpodella* sp. in this region, highlighting its potential as an emerging pathogen. Our findings provide preliminary evidence of human infection and underscore the urgent need for further epidemiological studies to evaluate the public health implications of *Colpodella* sp.

## 1 Introduction

*Colpodella*, a protozoan parasite, is increasingly gaining attention due to its close phylogenetic relationship with important apicomplexan pathogens, such as *Babesia* and *Theileria*. Accumulating research has demonstrated that *Colpodella* can cause opportunistic infections in humans and animals (1–5) and have been identified in potential ectoparasite vectors including ticks and flies (6, 7). Indeed, *Colpodella* sp. has been detected in at least nine tick species, including *Ixodes persulcatus*, *Rhipicephalus microplus*, *Dermacentor nuttalli Olenev*, *Haemaphysalis longicornis*, and *Hyalomma dromedarii* (8–12), highlighting the potential role of ticks as vectors for spreading the infection. Notably, two human cases of *Colpodella* sp. infection reported in China (from Heilongjiang and Yunnan Provinces) presented distinct clinical manifestations: one patient exhibited hemolytic anemia, while the other presented neurological symptoms, such as dizziness, gait disturbances, and headache. The scarcity of confirmed human cases limits our understanding of the organism’s clinical spectrum. Moreover, the atypical clinical presentations of *Colpodella* sp. infections, combined with limited awareness among healthcare professionals and diagnostic limitations, pose significant challenges for accurate diagnosis. Current diagnostic shortcomings include the absence of standardized primers for molecular detection and undefined antigen epitopes for serological testing, which may lead to misdiagnosis or underdiagnosis. These diagnostic challenges, coupled with the absence of systematic epidemiological surveys in China, hinder accurate assessment of infection prevalence and identification of risk factors associated with *Colpodella* sp. infections, thereby raising substantial public health concerns.

Our research group has previously identified *Colpodella* sp. DNA in animals, including domestic cats and dogs in Guiyang City, Guizhou Province (13), as well as in *Rhipicephalus microplus* ticks collected from Qiandongnan Prefecture, Guizhou Province (unpublished). Despite these findings, no human cases of *Colpodella* sp. infection have yet been reported in Guizhou Province, leaving the infection situation in the human population uncertain. This report documents the detection of *Colpodella* sp. in the blood of an ICU patient co-infected with adenovirus. Findings from this research will provide essential data for evaluating the potential public health threat posed by *Colpodella* sp. in this region, ultimately guiding future surveillance, diagnostic, and preventive strategies.

## 2 Case presentation

On May 21, 2024, a 28-years-old male patient from Qiandongnan Prefecture, Guizhou Province, was admitted to a tertiary care hospital with a primary complaint of recurrent fever lasting 1 week. Five days before admission, the patient had presented to a local hospital with fever (40°C–43°C), cough, and myalgia, receiving conservative treatment with acetaminophen for 3 days without any clinical improvement, and the fever persisted.

Upon admission, a routine exam showed a body temperature of 39.5°C, a blood pressure of 120/75 mm Hg, a pulse rate of 100 beats/min, and a respiration of 20 breaths/min. Lung auscultation revealed coarse breath sounds bilaterally, accompanied by wet rales. The laboratory tests are shown in Table 1. A routine blood test showed that the platelet count was decreased, as was the hemoglobin level, and the absolute lymphocyte count was also low. C-reactive protein levels were significantly elevated. Liver function tests showed decreased total protein and albumin. Urinalysis revealed positive urine ketone bodies, positive urine occult blood, and weakly positive urine protein characterization. Electrolyte analysis indicated demonstrated decreased sodium levels. A CT scan with three-dimensional imaging of the lungs revealed a partial solid lesion in the right lower lung, along with a small amount of fluid in the right thoracic cavity and interlobar fissure. The epidemiological history indicates that the patient, a farmer by occupation, reported no recollection of tick bites. He has not left his hometown in Qiandongnan Prefecture in the 6 months preceding the onset of illness and had no history of blood transfusions, but did report contact with companion animals. The initial diagnosis included fever, type I respiratory failure, pneumonia, thrombocytopenia, hepatic insufficiency, starvation ketosis, and electrolyte disorders.

**TABLE 1:** Laboratory tests of the patient.

Laboratory test	Test item	Results	Normal range (14–16)
Complete blood counts	WBC (/L)	5.18 × 10^∧^9/L	4.5–6.5 × 10^∧^9/L (14)
	RBC(/L)	4.25 × 10^∧^12/L	3.5–9.5 × 10^∧^12/L (14)
	Platelet count	91.0 × 10^∧^9/L	125–135 × 10^∧^9/L (14)
	Hemoglobin	128.0g/L	139−184g/L (14)
	Absolute lymphocyte count	1.08 × 10^∧^9/L	1.1–3.2 × 10^∧^9/L (14)
C-reactive protein		107.69 mg/L	0–5mg/L (15)
Liver function tests	Total protein	57 g/L	65–85g/L (15)
	Albumin	32.20 g/L	40–55g/L (15)
Urinalysis	Urine ketone bodies	2+	−(16)
	Urine occult blood	2+	−(16)
	Urine protein	+	−(16)
Electrolyte analysis	Sodium	133 mmol/L	135–145mmol/L (15)
	Potassium	3.54 mmol/L	3.5–5.5mmol/L (15)

–, negative; +, weakly positive; 2+, moderately positive.

On the first day of admission, the patient was administered antimicrobial therapy with piperacillin sodium-tazobactam sodium (Kefzol) 4.5 g (every 8 h). However, the patient’s symptoms showed no improvement, with persistent fever. The patient’s blood and sputum samples were analyzed by NGS on the second day of admission. The NGS results indicated that human adenovirus group B was detected in both blood and sputum. Subsequently, the patient received ganciclovir (0.25 g intravenously once daily) for antiviral therapy, supplemented with moxifloxacin (0.4 g intravenously once daily) to prevent secondary bacterial pneumonia. After 1 day of treatment, the patient’s temperature gradually decreased and his condition stabilized. After 1 week of treatment, the symptoms resolved completely, and the patient recovered well and was discharged from the hospital.

The blood sample was stored in liquid nitrogen and transported to the Research Laboratory Center, Guizhou Provincial People’s Hospital, on July 2, 2024, for further testing. The study was approved by the Ethics Committee of Guizhou Provincial People’s Hospital (Approval No.: 2023-077), in accordance with the medical research regulations of China. Specific PCR assays targeting common tick-borne pathogens, including *Borrelia burgdorferi*, *Babesia*, *Anaplasma*, and *Rickettsia*, as well as newly emerging tick-borne pathogens such as *Colpodella* sp., *Xue-Cheng virus*, and *Yezo virus*, were conducted for blood sample. Except for the PCR assay targeting *Colpodella* sp. in the blood sample, all were negative. After performing bidirectional sequencing and assembly, a 423 bp segment of the target 18S rDNA sequence was obtained. BLAST sequence alignment revealed a high similarity of this sequence to *Colpodella* sp.

**FIGURE 1:**
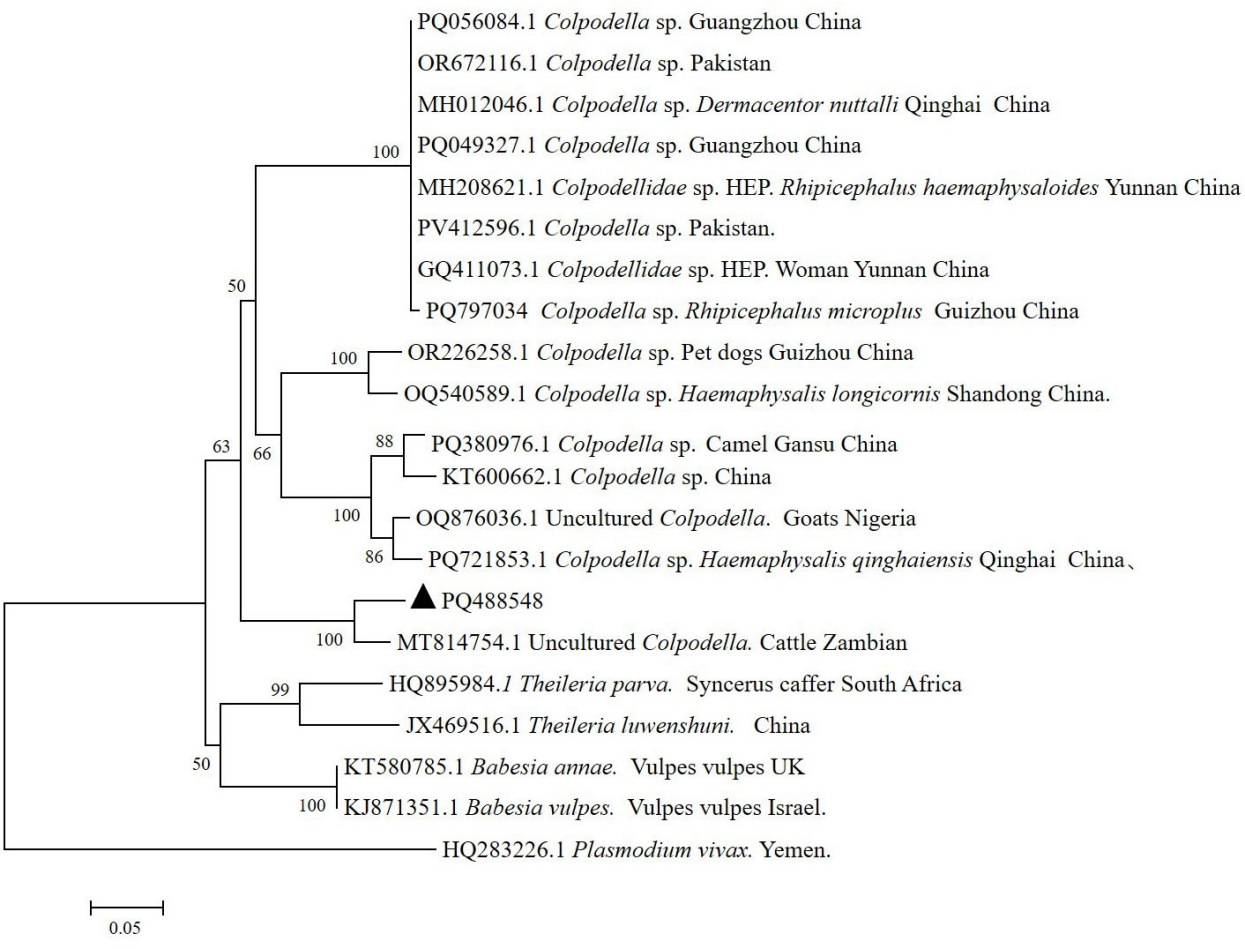
Phylogenetic analysis of *Colpodella* sp. infection in patient from Guizhou Province, China, based on a 423 bp fragment of the 18S rRNA gene. ▲:The 18S rRNA gene sequences of *Colpodella* sp. were obtained from patient. Maximum-likelihood method was used to construct a phylogenetic evolutionary tree and self-extension test 1,000 times for genetic evolutionary analysis.

The Successful sequencing and subsequent phylogenetic analysis of the positive amplicon revealed that the obtained sequence clustered within the same branch as an uncultured *Colpodella* from Zambian cattle (92.13% sequence identity). In contrast, the genetic sequences from *Colpodella* sp. (OR226258.1), detected in pet dogs from Guiyang, Guizhou Province, and *Colpodella* sp. (PQ797034), detected in *Rhipicephalus microplus* from Qiandongnan Prefecture, Guizhou Province, do not cluster within the same branch, indicating a more distant evolutionary relationship (Figure 1). This sequence has been submitted to GenBank with the accession number No. PQ488548.

## 3 Discussion

In this study, we observed the co-infection of adenovirus and *Colpodella* sp. in the blood sample of an ICU patient. Clinical analysis of the patient revealed severe complications, including type I respiratory failure, severe pneumonia, hepatic dysfunction, and starvation-induced ketosis, indicating an acute and critical systemic metabolic disturbance. Such multi-system dysfunction may severely impair host immune responses, increasing the susceptibility to opportunistic infections. Therefore, *Colpodella* sp. might have acted as an opportunistic pathogen contributing to this complex infection scenario. The patient resided in rural Qiandongnan Prefecture of Guizhou Province and was a farmer by occupation. The risk factor identified in their daily activities was frequent close contact with companion animals. Previously, our research group had detected *Colpodella* sp. in companion animals from Guizhou Province and had also identified DNA sequences of *Colpodella* sp. in the *Rhipicephalus microplus* collected in Qiandongnan Prefecture, where the patient lived. Given the patient’s close contact with companion animals, potential transmission pathways for the infection may include direct contact with infected animals.

Human infections caused by *Colpodella* sp. have been previously reported. Phylogenetic analysis conducted in the present study revealed considerable genetic divergence between this isolate and previously documented pathogenic human-infective strains of *Colpodella* sp., indicating potential differences in pathogenic properties. Notably, the most closely related genetic sequence identified in an uncultured *Colpodella* strain from Zambian cattle exhibited only 92.13% genetic identity. Therefore, the isolate examined in this study likely represents a genetically distinct *Colpodella* variant. This genetic uniqueness expands our understanding of *Colpodella* diversity and underscores the need for further genomic surveillance of *Colpodella* spp. to delineate species boundaries and assess their zoonotic potential. Moreover, this isolate exhibited relatively low sequence similarity and a distant phylogenetic relationship to previously described *Colpodella* sp. detected in *Rhipicephalus microplus* from Qiandongnan and companion animals from Guiyang City, further highlighting the considerable genetic diversity present within the genus *Colpodella.* Given the potential zoonotic transmission risk associated with *Colpodella* sp. it is advisable to enhance epidemiological surveillance to fully assess its public health implications.

Presently, there is no evidence indicating that infection by this *Colpodella* sp. isolate induces distinct clinical symptoms in the patient. Although *Colpodella* sp. was detected in the patient’s blood along with certain hematological abnormalities (decreased platelet count and hemoglobin levels), the patient exhibited no apparent hematological symptoms before or during hospitalization. Following a confirmed diagnosis of human adenovirus infection by next-generation sequencing (NGS) analysis of blood and sputum samples, and subsequent 8-day antiviral and antimicrobial therapy, the patient’s body temperature progressively returned to normal, clinical symptoms resolved completely. These observations strongly suggest that the clinical symptoms were primarily attributable to adenovirus infection. Furthermore, the *Colpodella* sp. isolated from the patient’s blood may exhibit limited pathogenicity and could represent an asymptomatic or transient bloodstream detection, particularly in immunocompromised individuals.

Nowadays, no specific treatment protocol has been clinically established for infections caused by *Colpodella* sp. According to previous literature, the anti-protozoal agent atovaquone, the macrolide antibiotic azithromycin and the tetracycline antibiotic doxycycline have all been reported to be clinically useful for managing *Colpodella* sp. infections (1, 3). From a pathogenetic perspective, the selection of atovaquone and azithromycin primarily is based on the phylogenetic relationship between *Colpodella* sp. and apicomplexan parasites such as *Babesia* spp. (17). In contrast, doxycycline is an antimicrobial agent typically used to treat bacterial infections. Its application in managing *Colpodella* sp. infections was based on the patient’s positive immunoglobulin G (IgG) antibody test against *Borrelia burgdorferi* sensu lato (sl), with a serum titer of 1:256, which led to a presumptive diagnosis of Lyme disease (3). Lyme disease, a bacterial infection, is frequently treated with doxycycline (18). In the present study, the patient achieved complete clinical resolution following antiviral therapy with ganciclovir and antibiotic therapy with moxifloxacin, without receiving antiparasitic treatment. This clinical outcome implies two potential possibilities: first, *Colpodella* sp. infection may be self-limiting, with spontaneous clearance achievable by the host’s immune mechanisms; second, the patient may have experienced a subclinical infection characterized by a pathogen load insufficient to elicit sustained clinical symptomatology.

This study has several limitations. Most notably, the inability to contact the patient precluded follow-up PCR testing to confirm whether the *Colpodella* infection had been cleared or persisted.

In conclusion, this study provides the first reported detection of *Colpodella* sp. in a patient within an intensive care unit (ICU). Factors such as the patient’s immunocompromised status, occupational exposure, and geographic context may have collaboratively contributed to this infection. These preliminary observations raise questions regarding potential transmission pathways, pathogenic mechanisms, and the possible threat of *Colpodella* sp. to human populations. Future research integrating epidemiological surveys, molecular biological analyses, and clinical research is required to further clarify the epidemiological characteristics and pathogenicity of *Colpodella* sp., thereby providing essential evidence for the development of public health preventive measures.

## Data Availability

The datasets presented in this study can be found in online repositories. The names of the repository/repositories and accession number(s) can be found in the article/supplementary material.
